# Nanoscale evolution of interface morphology during electrodeposition

**DOI:** 10.1038/s41467-017-02364-9

**Published:** 2017-12-19

**Authors:** Nicholas M. Schneider, Jeung Hun Park, Joseph M. Grogan, Daniel A. Steingart, Haim H. Bau, Frances M. Ross

**Affiliations:** 1 Department of Mechanical Engineering and Applied Mechanics, University of Pennsylvania, Philadelphia, PA 19104 USA; 2 Department of Materials Science and Engineering, University of California-Los Angeles, Los Angeles, CA 90095 USA; 3 IBM T. J. Watson Research Center, Yorktown Heights, NY 10598 USA; 4 Department of Mechanical and Aerospace Engineering, Andlinger Center for Energy and the Environment, Princeton University, Princeton, NJ 08544 USA

## Abstract

Control of interfacial morphology in electrochemical processes is essential for applications ranging from nanomanufacturing to batteries. Here, we quantify the evolution of an electrochemical growth front, using liquid cell electron microscopy to access unexplored length and time scales. During galvanostatic deposition of copper from an acidic electrolyte, we find that the growth front initially evolves consistent with kinetic roughening theory. Subsequently, it roughens more rapidly, consistent with diffusion-limited growth physics. However, the onset of roughening is strongly delayed compared to expectations, suggesting the importance of lateral diffusion of ions. Based on these growth regimes, we discuss morphological control and demonstrate the effects of two strategies, pulse plating and the use of electrolyte additives.

## Abstract (web summary)

Understanding structure evolution during electrochemical growth is crucial in materials processing and design of devices such as batteries. Here, the authors image copper during electrodeposition to provide strategies for controlling interface morphology.

## Introduction

The morphology that develops during electrochemical deposition determines the quality of electroplated coatings and the properties of porous structures, and can also be responsible for catastrophic failure during cycling of rechargeable batteries. Understanding how growth conditions drive the evolving interface toward either a planar or unstable morphology is therefore critically important in developing strategies to control the final structure. The governing physics of growth instabilities has been studied intensively. Linear stability models for the solid–liquid interface are well established for solidification and electrodeposition^1–3^. These analyses apply to quasi-steady-state conditions and generally do not account for transient phenomena^4^, occurring for example at the start of deposition, that can determine the future evolution. Constructing models for transient behavior is challenging because the critical stages, at the start of deposition or at a transition between growth regimes, are difficult to access experimentally with the required temporal and spatial resolution. The recently developed technique of liquid cell transmission electron microscopy (TEM) allows time-resolved nanoscale imaging of liquid–solid interfaces^5,6^. The ability to record movies during electrochemical deposition and correlate with electrochemical parameters^7–20^, as well as to control liquid geometry and quantify beam effects^19,21,22^, suggests that deposition physics can be accessed during these critical moments.

We therefore use liquid cell TEM to quantify transient growth front dynamics during electrodeposition at nanometer and tens of msec resolution. During galvanostatic deposition of copper, we obtain average measures such as the root mean square roughness and local measures such as the evolution of individual asperities. We find that growth initially follows the predictions of kinetic roughening theory then undergoes a transition to diffusion-limited behavior. However, this transition takes place more slowly than expected, suggesting a stabilizing effect of lateral diffusion. Based on the resulting picture of growth front evolution, we describe the role of the initial surface and discuss the use of two strategies for controlling morphology: current modulation and electrolyte additives.

## Results

### Growth front evolution

**Fig. 1:**
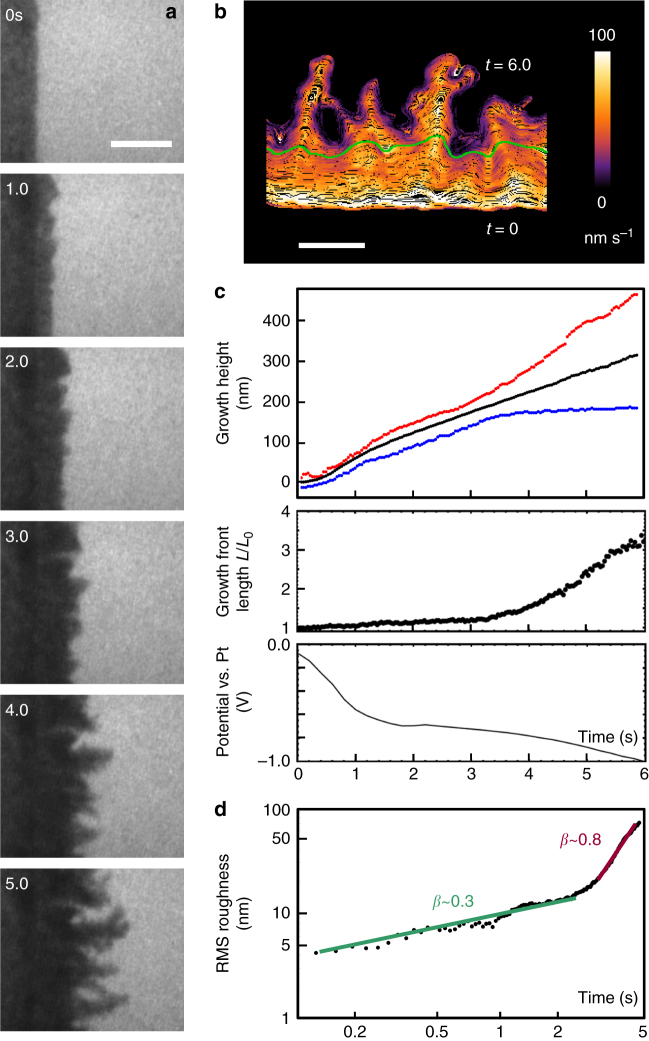
Electrochemical deposition of Cu at high growth rate. **a** Images recorded in bright-field conditions extracted from Supplementary Movie M1. The electrolyte was 0.1 M CuSO_4_ + 0.18 M H_2_SO_4_ with a total current through the cell of 400 nA. Times are shown in seconds since current flow began. Scale bar 300 nm. **b** Growth front position from movie frames color coded by normal growth speed. The transition time is indicated in green. Scale bar 200 nm. **c** Structural and electrical data as a function of time. The three graphs show the growth of height measured from the initial electrode edge (black = average; red = maximum; blue = minimum), the total length of the growth front measured from **b** and divided by the straight line length, and the measured potential. The growth height data yield an average growth rate of 55 nm s^−1^, equivalent to current density of 1550 A m^−2^. Note the transition in structure is at ~3.1 s. In the potential, the initial decrease is associated with geometry changes in the first 1 s as described in Supplementary Note 1; the later gradual decrease is associated with the development of growth front asperities. **d** RMS roughness of the growth interface shown as a log–log plot. Best-fitting straight lines and exponents are shown

**Fig. 2:**
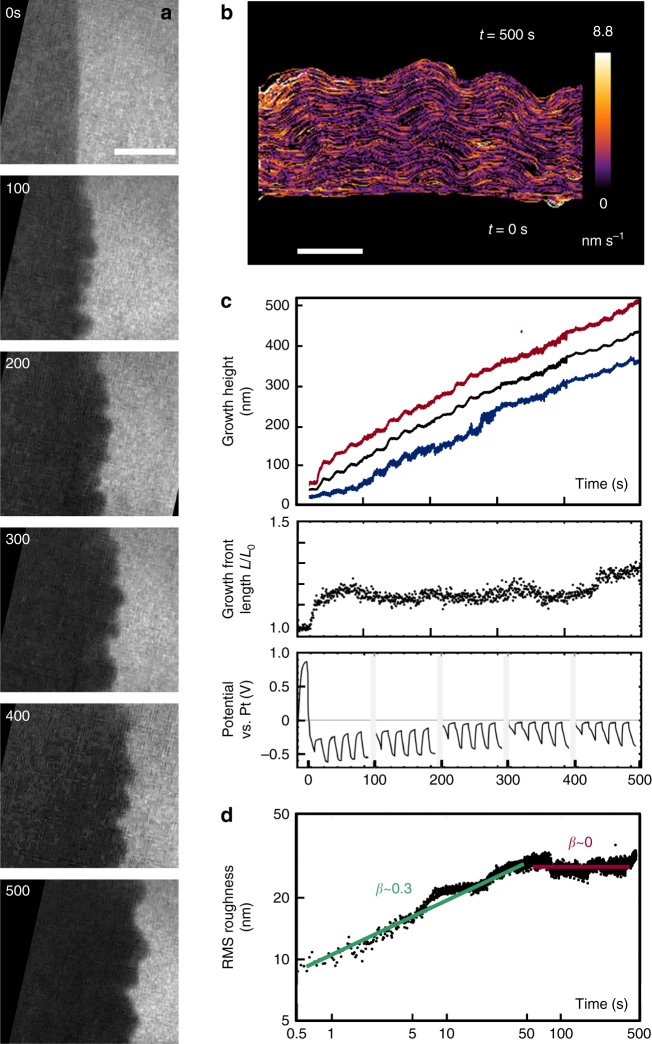
Electrochemical deposition of Cu at lower growth rate. **a** Bright-field images extracted from Supplementary Movie M2. The electrolyte is the same as in Fig. 1, but with cycles of 75 nA for 10 s/zero current for 10 s applied repeatedly after an initial cleaning cycle of 15 s. Time *t* = 0 indicates the end of the initial cleaning cycle. An image is shown then and after every five current pulses. Each set of five pulses takes 100 s and there is a longer interval between each set that is not included in the indicated time. Scale bar 300 nm. **b** Interface position from movie frames, color coded by normal growth speed. Scale bar 200 nm. **c** Structural and electrical data as a function of time: growth height (black = average, red = maximum, and blue = minimum), relative length of the growth front, and potential. In the potential, the initial peak is associated with the electrode cleaning cycle; the gray bars indicate the intervals between groups of five current pulses. The average growth rate during the on-time is 1.3 nm s^−1^, equivalent to current density 35 A m^−2^. The staircase shape of the growth front position reflects the growth-pause cycles. Slow etching (more than 10 × slower) retracts the growth front slightly during times of zero current (Methods). **d** RMS roughness of the growth interface shown as a log–log plot with best-fitting straight lines and exponents. The off time is included to reflect that diffusive behavior is governed by the total time

The evolution of an unstable growth front during galvanostatic copper deposition is shown in Fig. 1a and Supplementary Movie M1. A smoother morphology obtained during slower growth is shown in Fig. 2a and Supplementary Movie M2. In these experiments, growth takes place from an electrode edge (dark). After an initial period of three-dimensional growth (Supplementary Note 1, Supplementary Fig. 1), the growth front starts to extend laterally into the electrolyte (acidified copper sulfate; bright). Since the electrolyte is confined into a thin layer by electron-transparent windows above and below (Methods), this lateral growth has an overall two-dimensional (2D) geometry that is suitable for modeling. By extracting the interface position at each movie frame^23^, we can quantify the growth front evolution.

In Fig. 1, the 3D growth is complete by ~1 s (Supplementary Note 2, Supplementary Fig. 2) and the lateral growth front motion is visible. The front is initially fairly smooth, commensurate with the edge roughness of the lithographically defined electrode. However, ramified features evolve over a few seconds, separated by deep valleys where much less growth has taken place. The average interface position advances at a constant rate (Fig. 1c), as expected given the galvanostatic conditions. The rate measured (55 nm s^−1^) yields an average current density in the imaged region of 1550 A m^−2^ (Supplementary Note 2). The experiment only records growth in a small area of the electrode. However, we expect growth to be similar in the imaged and nonimaged areas, because the average current density in the imaged region is similar to a device-averaged current density of 1450 A m^−2^, obtained by dividing the total current (400 nA) by the total growth area (electrode perimeter, 1380 µm × growth front height, nominally 200 nm).

In Fig. 1b, sequential growth front profiles are plotted with colors that indicate the normal growth velocity at each time and location. As growth proceeds, the velocity ceases to be uniform, with higher velocities at the peaks and little or no growth at the bottom of the developing valleys. Two regimes are apparent in Fig. 1b, c. When *t *< 3 s, the minimum and maximum growth height, or distance of the growth front from the initial electrode edge, both advance. After this transition time, the locations of minimum height (valleys) become essentially stationary and the maximum height grows more rapidly. The total length of the interface (Fig. 1c) increases slowly at first and then more rapidly after the transition time.

Growth models often characterize surface morphology with the root mean square (RMS) roughness, rather than these types of spatially resolved features^24–27^. To enable comparison with these models, Fig. 1d shows RMS roughness vs. time. The roughness appears to scale as a power law in time with two distinct exponents, indicated on the graph, that match with the two regimes in Fig. 1c. Before the transition time (we label this as the planar regime), the roughness grows slowly with an exponent of 0.3, but after the transition time (the ramified regime), the exponent increases dramatically; rapid growth of ramified asperities and no growth at the bottom of the valleys produce an exponent around 0.8.

### The transition between growth regimes

The planar and ramified 2D growth regimes visible in Fig. 1 appear quantitatively consistent with two growth models. The planar regime is consistent with growth by random arrival of material, i.e., kinetic roughening^4,25^. This causes the surface to roughen stochastically (at early times; see below). Kinetic roughening alone is expected to produce an initial exponent of 0.5^25^. Forces that smooth the surface, such as atomic diffusion, are expected to yield values <0.5^26^. The planar regime in Fig. 1 with exponent 0.3 is, thus, consistent with kinetic roughening plus surface smoothing. However, the ramified regime, dominated by spatial variation in current density and with deposition only at the peaks, is consistent with diffusion-limited control. Such growth occurs when ions from the bulk solution follow the path of least resistance to the highest local asperity^27,28^, while low points along the front are screened or deprived of incoming ions. The expected exponent for such a regime is between 0.5 and 1.0, consistent with our measured value of 0.8.

To test the idea that kinetic roughening dominates at early times, we examine the growth in Fig. 2. This growth was designed to minimize diffusion limitations to reveal the early regime; the total current was reduced by 5x and was also interrupted for 10 s after every 10 s to allow diffusion to replenish the depleted electrolyte adjacent to the growth interface. The resulting RMS roughness (Fig. 2d) increases with exponent 0.3, similar to the planar regime in Fig. 1. It then reaches a time-independent plateau with exponent ~0. At this time, growth is occurring at both the peaks and valleys at approximately the same rate; the peak-valley separation approaches a constant (Fig. 2b, c). Diffusion-limited kinetics is not observed. Instead, the whole evolution appears to follow the behavior expected for kinetic roughening, in particular ballistic deposition, where models predict an RMS exponent below 0.5 followed by a constant roughness^25^. We presume that a constant roughness was not seen in Fig. 1 because that growth entered the diffusion-limited regime first.

Given this transition from kinetic roughening to diffusion-limited models, we can calculate the time at which we would expect diffusion-limited physics to take control. In a simple approximation, of planar, constant current growth with no ion transport other than diffusion (Supplementary Note 3), this is the Sand Time^29^. At this time the ion concentration at the interface has dropped to zero; the applied current can no longer be supported by the copper deposition reaction, so the system responds by changing its surface morphology and growing under diffusion-limited conditions^30–33^. (The system could respond in principle by changing the potential to a value that supports another electron transfer reaction, but such changes are not seen in the potential, Fig. 1c). For the parameters in Fig. 1, we calculate the planar ion concentration as a function of time and position (Supplementary Movie M3, Supplementary Fig. 3) to obtain a Sand Time of below 0.1 s, well below the transition time at which the experiment shows characteristics of diffusion-limited growth. Furthermore, we would expect the onset of diffusion-limited growth at the Sand Time to involve the rapid development of asperities, as well as a change in potential^30^ to more negative values in a galvanostatic experiment. (A potential change is expected since diffusion-limited growth takes place primarily at the tips of the asperities and hence at higher local current density). In Fig. 1c, a plot of the growth front length (a measure of growth front area and roughness) shows the expected increase, but changes are gradual and only start at 3–4 s. The potential shows similarly gradual changes at this time (the steeper change before 1 s is associated with the initial 3D growth, see Supplementary Note 2). The gradual nature of these changes is consistent with the morphology of the actual growth front position at the transition time (Fig. 1b); the growth front length does not increase suddenly because the fluctuations that form the asperities are present even before the transition time.

**Fig. 3:**
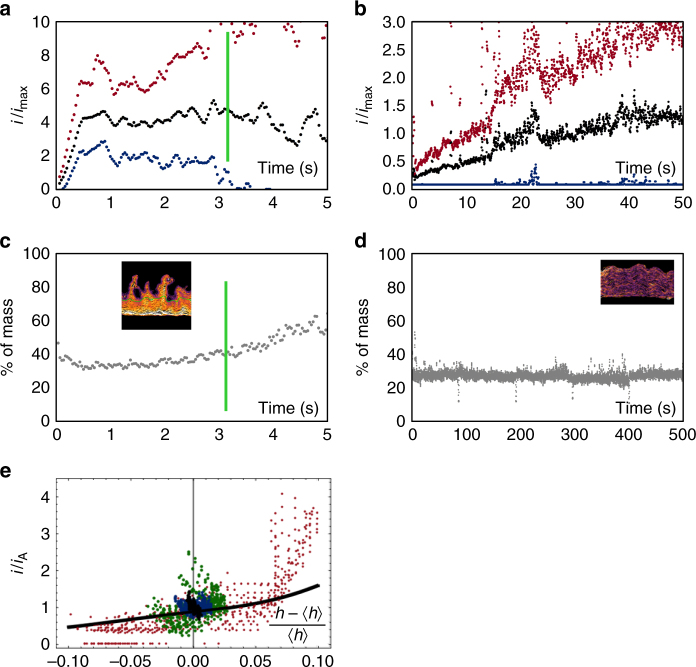
The pattern of current flow at the growth front during deposition. **a**, **b** Experimental maximum, minimum, and average current density obtained from the line profile plots in Figs 1b and 2b, respectively. The values are normalized by dividing by the maximum current that could be sustained by planar diffusion at that time given that current* i* has already flown for time *t*, where *i* = 1550 and 35 A m^−2^ for **a** and **b**, respectively. **c**, **d** Percent of mass added to the top quartile of points during the experiments shown in Figs  1 and 2, respectively. Noise in **a**–**d** reflects errors in aligning adjacent movie frames. **e** Relationship between normalized current density and deviation from mean height for diffusion-limited growth of a sinusoidal profile calculated from ref. ^34^ (black line). Growth data from Fig. 1 are shown at* t* = 0.0 s (black), 2.0 s (blue), 3.0 s (green), and 3.8 s (red)

The Sand Time thus appears to be necessary but not sufficient for the onset of diffusion-limited growth. Since the Sand Time is calculated for planar geometry, a simple explanation is that the nonplanar geometry visible at the growth front is associated with lateral variations in ion concentration, and hence lateral ion diffusion that can circumvent the limitations of planar diffusion. Consistent with the presence of lateral diffusion is Fig. 3a, which quantifies the fluctuations in local current density in Fig. 1. This graph shows that even before the observed transition time, there are some points along the growth front where material is added at a higher rate than could be sustained by planar diffusion. Mechanisms such as surface diffusion would blunt asperities, not add material at such locations. Although ion concentrations are not measured directly here, the presence of lateral diffusion is also consistent with interferometry data at larger length scales^30^ that shows ion concentrations between asperities.

More detailed analysis of the development of asperities in the diffusion-limited regime requires the coupled relationship between geometry and current density. This has been solved for diffusion-limited growth of a sinusoidal surface^34^ to derive an expression (Fig. 3e) relating the deviation from the average current density, *i*/*i*_A_, to the deviation from the mean height, $\frac{{h - \left\langle h \right\rangle }}{{\left\langle h \right\rangle }}$. Here, *i* is the current density, *h* the interface height at a point, and, *i*_A_ and $\left\langle h \right\rangle$ the average values. Figure 3e also shows the experimental data. Before the transition, the data are strongly clustered with little deviation in height and all velocities similar. After the transition, both height and velocity spread, somewhat consistently with the expression in ref. ^34^. Furthermore, Fig. 3c characterizes how strongly the current is focused at the asperities by showing a so-called growth inequality, or relative growth rate of the highest quartile of points along the growth front. At early times, the highest 25% of the growth front consumes ~30% of the incoming ions, but after the transition, the top 25% consumes over 50%. This trend is also consistent with expectations from diffusion-limited growth, and it may be possible to use such data in models that include geometries that are more complex than sinusoids.

For the slower growth, the growth front profiles show more uniformly distributed current density (Fig. 2b). The potential (Fig. 2c) follows a complex pattern because of the pulsed current and the additional recovery time between the groups of pulses, but is overall consistent with the expectations for current pulses shorter than the Sand Time^35^. The growth front length remains fairly constant (Fig. 2c) and the mass inequality is low throughout deposition (Fig. 3d). The uniform current density, assuming Butler–Volmer-type kinetics, implies uniform, nonzero ion concentration at the interface. Indeed, a calculation of the ion concentration as a function of time and position (Supplementary Movie M4, Supplementary Fig. 3) confirms that the ion concentration at the growth front is not expected to reach zero. It is therefore unsurprising that diffusion-limited characteristics are not seen in this experiment. However, there are still points along the interface where material is added at a rate higher than that which could be sustained by planar diffusion (Fig. 3b), suggesting the presence of lateral diffusion.

Summarizing Figs 1–3, the front initially evolves consistent with kinetic roughening models. It can continue along the kinetic roughening path, with roughness eventually saturating, as in Fig. 2. Alternatively, the asperities that have formed can transition into a ramified growth front. But even before this transition, there are gradual changes in the surface area (and potential), the spread of local growth velocities, and the mass inequality. The distinguishing feature of the transition time appears to be that the current density at the lowest points of the growth front approaches zero. The transition occurs later than expected from planar diffusion, which we attribute to the stabilizing effect of lateral diffusion. After the transition time, the current distribution continues to become more unequal. Although growth has stopped at the valleys, the maximum current density does not increase very rapidly, presumably due to tip-splitting events (Fig. 1b includes an example) where perturbations at the tip undergo roughening and then diffusion-limited growth^33^.

### Control of growth morphology

**Fig. 4:**
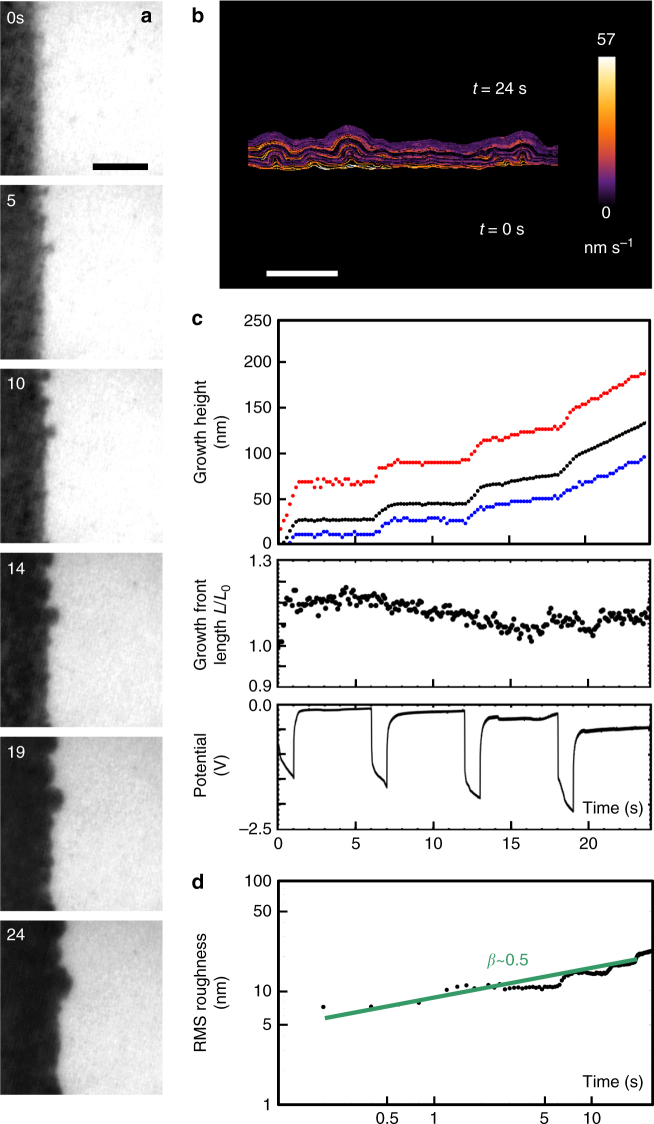
Electrochemical deposition of Cu under pulse-plating conditions. **a** Images recorded in bright-field conditions extracted from Supplementary Movie M5. Deposition took place in cycles consisting of total current 400 nA for 1 s and then zero for 5 s. The electrolyte was the same as in Fig. 1. Times are shown in seconds since current flow began. Scale bar 300 nm. **b** Interface position from movie frames, color coded by normal growth speed. Scale bar 200 nm. **c** Structural and electrical data as a function of time: growth height (black = average; red = maximum; and blue = minimum), relative length of growth front, and potential. The average growth rate, including off time, is 13 nm s^−1^. This is equivalent to 340 A m^−2^ time-averaged current density, or 2040 A m^−2^ in the pulses. **d** RMS roughness of the growth interface shown as a log–log plot. The best-fitting straight line and exponent are shown

If the ramified features that dominate late-stage morphology are initiated during the kinetic roughening regime, growth front planarity cannot be improved by starting from an especially smooth surface. Other strategies must be used. Pulsed (interrupted) growth is a well-known approach to avoid the transition into diffusion-limited kinetics, forming smooth layers even for current densities high enough to cause instabilities if applied continuously^36^. Figure 4 and Supplementary Movie M5 show deposition using short pulses of a high current similar in magnitude to that in Fig. 1. The 1 -s on-time was chosen to be shorter than the measured transition time in Fig. 1. The 5 -s off time was chosen to allow diffusion to replenish ions near the electrode, based on a calculation of the ion concentration during pulsing. Ramified growth does not develop. The maximum, minimum, and average height show little change in their spread during growth, the local growth velocity is fairly uniform, and the growth front length does not increase with time (Fig. 4b, c). The RMS roughness average growth exponent is ~0.5 (Fig. 4d), similar to that expected for random uniform deposition, and the potential in Fig. 4c is consistent with current pulses shorter than the Sand Time. However, the calculated Sand Time for the current used is only 0.05 s, suggesting that instabilities should be expected during every pulse. The behavior is therefore similar to that of Fig. 1 in the sense that the transition to diffusive growth is slower than expected; this is of possible relevance to the design of pulse-plating schemes^36^.

**Fig. 5:**
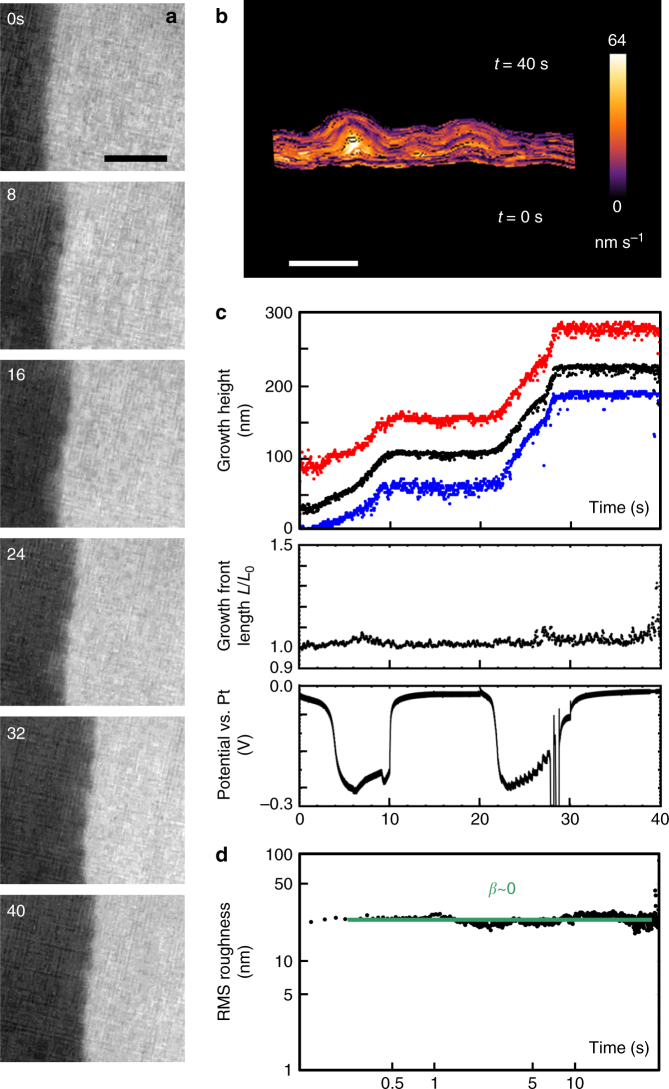
Electrochemical deposition of Cu in the presence of an additive. **a** Images recorded in bright-field conditions extracted from Supplementary Movie M6. Deposition took place in the same electrolyte of Fig. 1, but with saturated PbSO_4_. A total of 300 nA was applied for 10 s, 0 nA for 10 s, and then repeated. Times are shown in seconds since current flow began. Scale bar 300 nm. **b** Interface position from movie frames, color coded by normal growth speed. Scale bar 200 nm. **c** Structural and electrical data as a function of time: growth height (black = average; red = maximum; and blue = minimum), total length of growth front, and potential. The average growth rate in the pulses is 14 nm s^−1^ and zero between pulses. This is equivalent to 380 A m^−2^ in the pulses. **d** RMS roughness of the growth interface shown as a log–log plot. The best-fitting straight line and exponent are shown

Additives comprise another strategy to control growth morphology^37^. Figure 5 and Supplementary Movie M6 show growth in an electrolyte with PbSO_4_ additive^38^. Based on a calculation of the ion concentration as a function of time and position for the experimental current density (Supplementary Fig. 3), the ion concentration at the growth front is not expected to reach zero in this experiment. Growth indeed shows fairly constant local growth velocities, constant peak height, constant growth front length, and potential consistent with current pulses shorter than the Sand time. The average growth exponent is only 0.02 and some initial smoothing is even possible. We hypothesize, with support of ex situ roughness measurements (Supplementary Note 4), that Pb acts as a surfactant that slows the reaction rate and allows surface diffusion to smooth the surface.

## Discussion

It is clear that the developing spatial and temporal pattern of current flow during deposition can be quantified using liquid cell TEM, achieving a resolution that complements interferometric techniques^30^. During deposition at different current densities, asperities form and initially behave consistent with kinetic roughening. At high current density, the pattern of growth then changes. There is a transition time where the asperities rapidly increase in amplitude. The transition time is marked by zero growth in the valleys and is delayed compared to expectations from calculations based on average parameters. But even before the transition time, hints of the upcoming instability are present in terms of the increasing inequality in the way that material is added to points along the growth front. Local growth velocity measurements point to the importance of lateral diffusion of ions, even at times before the transition time.

Controlling growth front roughness is important in battery cycling and in applications of electrodeposited films. Unsurprisingly, the liquid cell data show that instability can be prevented (at least, over the experimental time frame) by reducing the growth rate. The asperities still form with the same initial kinetics as seen at the higher growth rate, but no transition to instability is seen and the growth front instead tends toward a constant roughness. However, in applications such as battery charging, it is important to maintain a smooth morphology even at rapid growth rates. The experiments suggest that an initially flat surface may not help to achieve this aim, because asperities initially appear and grow in a similar way irrespective of whether severe roughness develops eventually. Instead, the in situ growth experiments illustrate the effects of two other strategies, the use of additives and pulse deposition, in changing the evolution of the growth front. We suggest that quantitative understanding of early-stage growth will lead to the development of other strategies to create specific morphologies in nanofabrication or control processes such as battery cycling.

## Methods

### Geometry of the liquid cell and deposit

Experiments were carried out with the Nanoaquarium^39,40^. This is a wafer-bonded liquid cell, made up of two chips, each with an electron-transparent viewing window in the center. Imaging takes place through the liquid layer between the two windows. An oxide spacer keeps the chips separated from each other by nominally 200 nm and seals the edges. On the upper chip, two ports allow the electrolyte to be introduced into the volume between the two chips.

Within the liquid cell, there are four thin-film Pt electrodes, two of which overlap the electron-transparent window. The electrodes are 25 -nm polycrystalline Pt on a 5 -nm Ti adhesion layer and are deposited by thermal evaporation followed by lift-off patterning. The geometry and the region visible in situ are shown in Supplementary Fig. 1. Note that the working and pseudo-reference electrodes are exposed to solution both within the viewing window and outside it. Each of these electrodes has an exposed surface area of 2.4 × 10^−8^ m^2^ and a perimeter of 1380 µm; the closest separation between them is 50 µm. The counter electrode has a total exposed area of 2.9 × 10^−7^m^2^ and is 1500 µm from the working electrode.

The nominal height of the liquid cell channel is set at 200 nm by the silicon oxide spacer, although bulging increases the liquid height in the center of the window. Calculations of membrane deflection, Supplementary Fig. 1c, show a nearly linear increase in liquid layer thickness from 200 nm to 300 nm over the first 2 µm as one moves from the silicon edge toward the center of the device (assuming a device at 1 atm of pressure, and calculated using the membrane deflection solution^41^).

Within this geometry, growth proceeds as shown in Supplementary Fig. 1e, based on the intensity analysis in Supplementary Note 1. Current flow first drives Cu nucleation and growth over the whole area of the working electrode. Depletion of the solution above the working electrode and blocking of incoming ions slow down the growth rate in the interior; at the edges, growth reaches the upper Si_*x*_N_*y*_ surface rapidly (the time required is 1 s for Fig. 1, as shown in Supplementary Fig. 2e). Continued growth at the edges is essentially two dimensional with thickness equal to the liquid height.

### Electrochemical measurements

The electrolyte used in Figs. 1, 2, and 4 was 0.1 M CuSO_4_ + 0.18 M H_2_SO_4_, made from aqueous solutions of each component prepared by dissolving 99.99% purity chemical in doubly deionized water. For Fig. 5, saturated PbSO_4_ (0.15 μM) was added.

In Figs. 1, 2, and 5, three-electrode setup was used, while in Fig. 4, two electrodes were used. The electrodes were controlled by a Gamry potentiostat. Galvanostatic experiments were carried out with total current through the cell in the range of 20 nA to 1 uA. A benefit of constant current techniques is that the ohmic drop due to solution resistance is constant. This is especially important for the liquid cell with its limited electrolyte volume. Calculations of current density and resistance in the electrode and electrolyte confirm that temperature rise due to ohmic heating is negligible.

### Image acquisition and reproducibility

The interface morphology evolution was imaged at 30 images per second in a Hitachi H-9000 TEM at 300 kV using bright-field imaging conditions. The field of view in the movies is 1.85 × 1.4 µm. The beam intensity was set at a low level to minimize radiolysis effects. Furthermore, the liquid thickness was chosen to be relatively large, several hundred nanometers to better replicate bulk physics. (This is greater than the nominal separation between wafers because there is some bulging of the windows due to the pressure difference). These imaging conditions result in noisy frames with little detail within the deposited film, but still resolve the growth front well enough to track and analyze.

To test the reproducibility of the liquid cell observations, 94 separate deposition experiments yielded consistent local growth front propagation speeds (Supplementary Note 2, Supplementary Fig. 4) and current densities above 300 nA consistently formed ramified growth fronts, although detailed analysis of growth exponents was not carried out for all the experiments.

### Etching during experiments

Copper may be oxidized by reactive species in the solution, especially, during off-times in pulse plating. In Fig. 2 (10-s periods at 75 nA of total current), the average interface velocity is 1.3 nm s^−1^. During pauses (10 s at zero current), the average interface velocity is −0.14 nm s^−1^. Interface etching therefore does occur, but is an order of magnitude slower than the slowest deposition rates analyzed.

### Electron beam effects

Electron beam effects can play a strong role in liquid cell electron microscopy. The beam-sample interactions produce molecular and radical products such as hydrogen, oxygen, and hydrated electrons that lead to beam-mediated phenomena. The beam current was kept low (0.01–0.15 nA) to ensure that electrodeposition dominates beam-induced deposition in the experiments. Irradiating the solution for several minutes (compared to the tens-of-seconds duration of electrodeposition), showed negligible growth of nanocrystals at the dose rates used. However, beam effects cannot be eliminated completely and often depend on the electrochemical conditions^12,21,22^; Supplementary Movie 1 shows visible beam-induced deposition 10 s after the end of the experiment in Fig. 1.

### Ion concentration calculations

Numerical integration of the solution of the diffusion equation for the Cu^2+^ concentration as a function of time and distance from the growth front was performed for the experimental parameters in each experiment. The initial concentration C(*x*, 0) = 0.1 M. For the rapid deposition rate in Fig. 1 (calculation shown in Supplementary Movie M3), the current density was applied as a Neumann-type boundary condition at *x* = 0 of constant current until the concentration reached zero at *x* = 0. The wall concentration was then transitioned to a Dirichlet boundary condition, and held at 0 for the remainder of the calculation. For the other calculations (one of which is shown in Supplementary Movie M4), the current density was applied as a time-dependent Neumann-type boundary condition at *x* = 0 with square wave in time. The calculation neglects motion of the growth front. Note the tens-of-micrometer length scale of these movies, in comparison with the motion of the growth front of a few hundred nanometers in Figs. 1–5.

### Data availability

The datasets analyzed during the current study are available from the corresponding author on reasonable request.

## Electronic supplementary material

Supplementary Information

Description of Additional Supplementary Files

Supplementary Movie 1

Supplementary Movie 2

Supplementary Movie 3

Supplementary Movie 4

Supplementary Movie 5

Supplementary Movie 6
