# Supplementary material for: Nanoscale evolution of interface morphology during electrodeposition
Source: Nat Commun. 2017 Dec 19;8:2174. doi: 10.1038/s41467-017-02364-9 (PMC5736733; doi:10.1038/s41467-017-02364-9)
Supplement: Supplementary file 1 — Supplementary Information [file 41467_2017_2364_MOESM1_ESM.pdf]

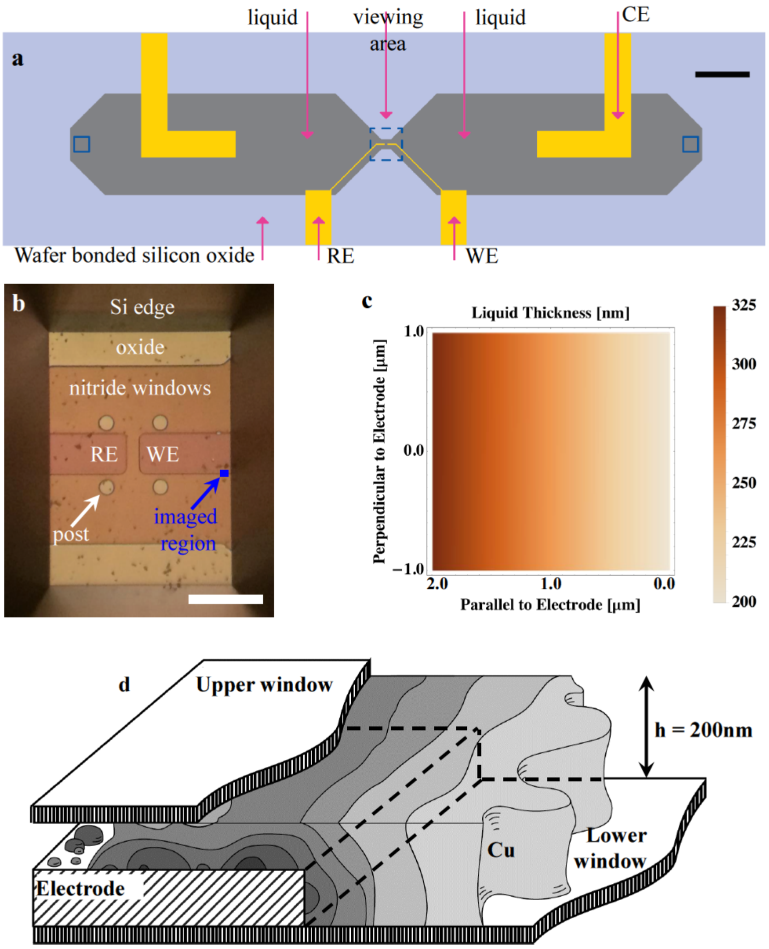

Supplementary Figure 1 Geometry of the electrochemical system

a Nanoaquarium electrode configuration. The gray area represents the nanochannel where the solution is in contact with the Si_xN_y and electrodes. The electrodes are labeled as used in Figures 1-5: WE, RE and CE are the working, pseudo-reference and counter electrodes respectively. Scale bar 500μm. **b** Light microscope image of the central area in **a** showing the viewing window in an (unfilled) device. The blue square shows the area imaged in all TEM experiments. The posts help control a minimum window separation. Scale bar 20μm. **c** Calculated liquid thickness in the TEM imaged region. The liquid thickness increases from 200nm to 325nm over a distance of 2μm due to membrane bowing. **d** Illustration of Cu growth geometry. Nucleation, coalescence and growth at the electrode edges extends to the top of the liquid layer, followed by growth perpendicular to the electrode at a constant thickness h. Lighter gray indicates later growth. The electrode height is exaggerated. The beam direction is vertical.

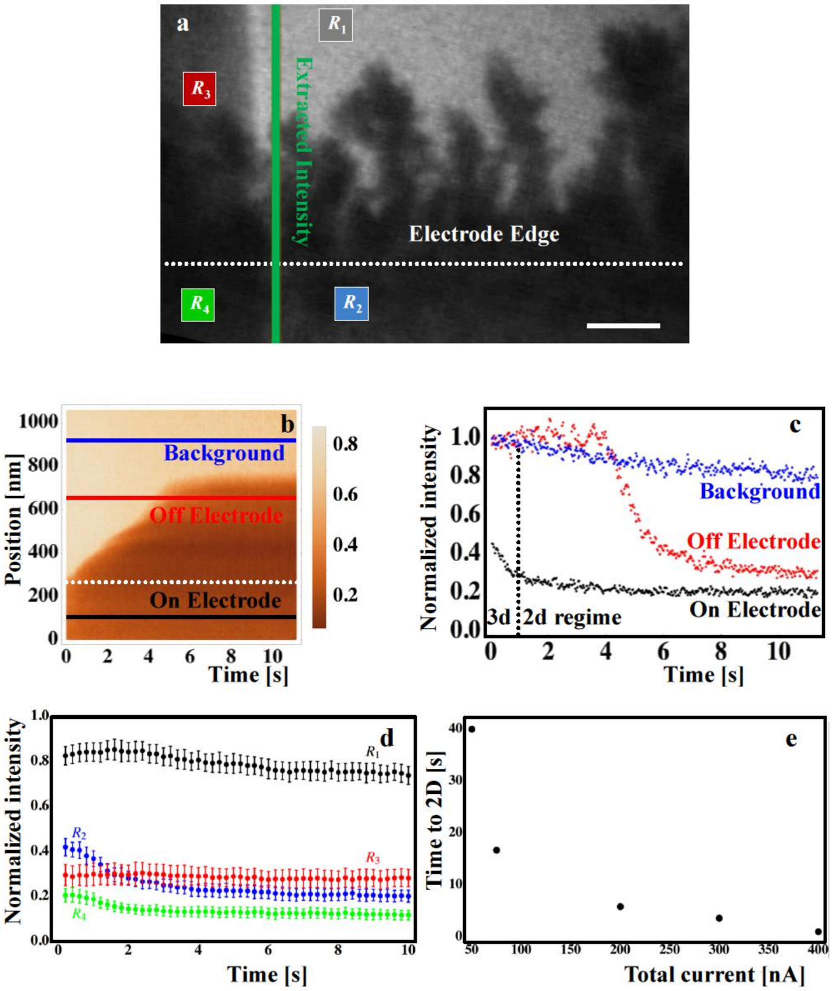

Supplementary Figure 2: Intensity measurements and the 2D growth regime

a Sample frame from Video M1 showing the location of a measurement of an intensity profile (green vertical line) further analyzed in **b**, **c**, and four boxes at which detector saturation was further analyzed in **e**. The white dotted line represents the electrode edge location. The measurement is averaged over 10 pixels width to reduce noise. Scale bar 250nm. **b** Contour plot of intensity along this line vs. time. The electrode edge (dotted white line) and two locations (black, red) are indicated. After the growth front has passed each point the intensity appears fairly constant, supporting the existence of a 2D growth regime. **c** Intensities at the black and red locations normalized by the background intensity. The time at which the growth has become 2D is taken to be 1s (dotted line). Note that the background intensity normalized by the t=0 intensity (blue) shows a slow decrease in the electron beam intensity during the experiment. **d** Test of detector saturation by plotting the mean intensities of the regions identified in **a**. Error bars show the standard deviation. **e** Deposition time until growth enters the 2D regime using the method in **c**.

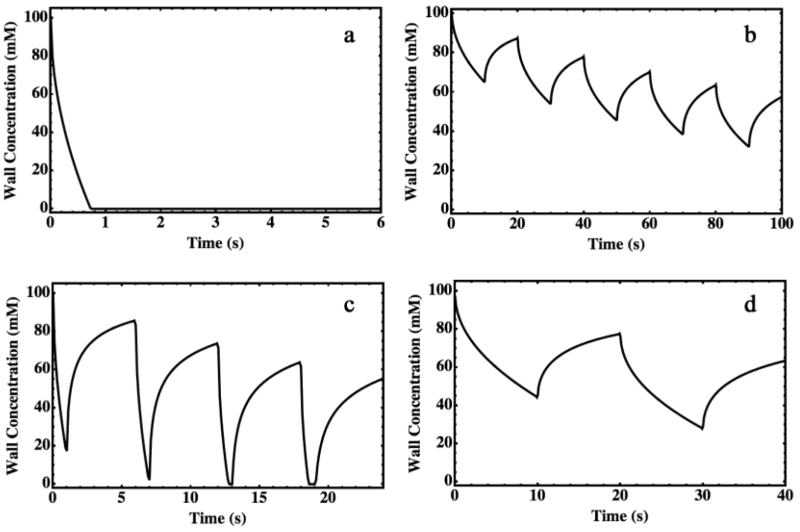

Supplementary Figure 3: Copper ion concentrations calculated from numerical solution of the diffusion equation.

a Concentration vs. distance from the growth front plotted at selected times for the simulation in Video M3. The times shown are 0, 0.001, 0.01, 0.05, 0.09 (the Sand time, in red), 0.2, 0.5, 1, 2, 3, 4 and 5 sec. Note the length scale in micrometers. The blue arrow shows the direction of time. **b** Concentration at the growth front vs. time for the same simulation. Note that the concentration reaches zero at the growth front at 0.09s. **c** Concentration at the growth front vs. time for Video M4. **d** Concentration at the growth front vs. time for a simulation using the current in experimental Video M6 but not including any effects of the Pb additive.

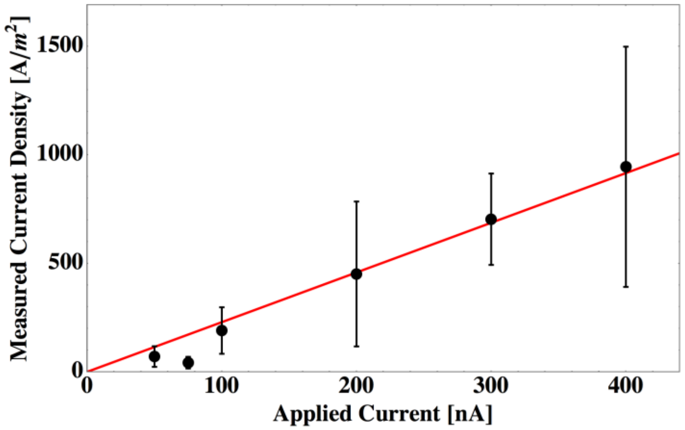

Supplementary Figure 4: Measured current density, calculated from average growth velocity in the 2d regime, for a total of 94 separate Cu deposition experiments from plain solution. The best-fit line through the origin has gradient 2.3×10^9 m^{-2}.

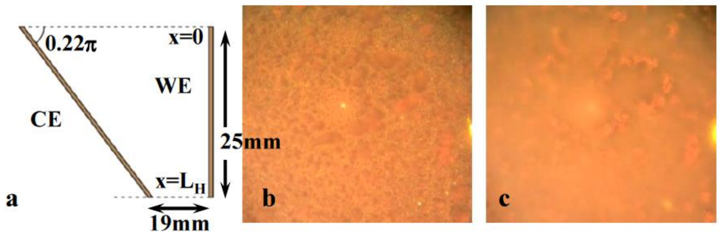

Supplementary Figure 5: **a** Schematic of Hull Cell design. The CE was coated at low current density with Cu to act as a Cu source. **b, c** Illustrative example of substrate in focus **b** compared to tips of dendrites in focus **c** at the same location. Images were measured along the mid-width of the WE to avoid edge effects.

Supplementary Table 1

Physical constants of species relevant to the experiments in an electrolyte containing 0.1 M $CuSO_4$ + 0.18M H_2SO_4

	Cu^{2+}	SO_4^{2-}	H^+
t_j	0.03	0.15	0.82
**z**	2+	2-	1+
**D**	0.71 x 10^{-9} m^2 s^{-1}	1.065 x 10^{-9} m^2 s^{-1}	9.312 x 10^{-9} m^2 s^{-1}
**c**	0.1M	0.280 M	0.360 M

.

Supplementary Table 2

Calculated Sand Time in a two dimensional channel with electroosmotic flow

**Current Density [A m^{-2}]**	**Slip velocity toward WE, 30 [μm s^{-1}]**	**Zero slip velocity**	**Slip velocity away from WE, 30 [μm s^{-1}]**
**100**	22.6 s	23.1 s	22.7 s
**1000**	0.237 s	0.238 s	0.237 s

Supplementary Notes

1 The two-dimensional growth regime

The data analyzed in Figures 1-5 is obtained from the two dimensional growth regime. To verify that growth reaches and then maintains a constant thickness (in the beam direction), in Supplementary Figure 2 we measure image intensity during growth. The intensity at each point in a bright field image provides an estimate of the projected atomic number density multiplied by material thickness. A darker (lower intensity) region represents more or denser material in the beam path. The intensity profile in (b, c) supports the hypothesis that the deposit reaches a constant thickness (h in Supplementary Figure 1d) then continues to grow laterally at that thickness. For example, the black data depicting intensity on the electrode drops quickly as Cu is deposited, almost all within the first 1 s, then remains almost steady suggesting no further Cu is added at this point. Off the electrode, the intensity drops after a delay as the growth front propagates. To confirm that the sensor is not saturated during this type of analysis we show in Supplementary Figure 2d the intensity vs. time at different points in the image. The region where silicon and electrode overlap shows the lowest intensity value, implying that the sensor is not saturated in other regions.

To estimate the time at which growth becomes two-dimensional, we create graphs akin to Supplementary Figure 2c for each current. The time to 2D is taken as the time at which the intensity at the electrode edge approaches its minimum value. The time falls rapidly with current, Supplementary Figure 2e. In particular, the time obtained in Video 1 is ~1s. This is consistent with the behaviour of the potential in Figure 3c. The transition from 2D growth to 3D growth implies a change in the effective area of the electrode, from an initial value of 2.4×10^{-8} m^2 to a value of perimeter × liquid height, 1380 µm × 200nm, or 3×10^{-10} m^2. The increased current density in the galvanostatic experiment requires a more negative potential to drive it, based on Butler-Volmer kinetics. This is consistent with the change in potential to a more negative value seen in the first ~1s in Figure 3c.

As an independent measurement of Cu thickness in the 2d regime, we use the intensity in the bright field images. This requires calibration of the change in intensity for a given increment of atomic number density thickness. As a first approximation, the intensity decrease (attenuation) is directly proportional to the sum of atomic number Z multiplied by thickness, integrated along the beam path. The electrode (25nm Pt on 5nm Ti, average Z=68.7) provides the necessary data to convert intensity change into mass thickness. We measure the intensity drop at a given location (taking care to normalize the images when beam conditions vary) as the growth front passes across this location, and scale by the ratio of Z for Cu and Pt/Ti. The result is an average Cu thickness of 122nm, which is reasonable given the height of the device defined by the lithography as 200 nm (and that this calculation relies on the accuracy with which the Pt thickness is known).

2. Local and global current densities and growth uniformity

The analysis of the Sand time assumes knowledge of the current density at the growth front in the 2D regime. Because of the complex liquid cell geometry it is important to examine the distribution of deposited material and relate the current density measured from the videos to the total applied current.

There are several ways one might *a priori* estimate current density. Initially one can assume that current flows over the whole working electrode area, 2.4×10^{-8} m^2. A uniform distribution yields, for example at total current 400nA, a current density of $400 \times 10^{-9}/2.4 \times 10^{-8} = 17$ A m^{-2}.

However, as shown above, the electrode area rapidly becomes screened and growth then occurs only at the edge of the electrode. In this 2D regime, the growth area A is the product of the electrode perimeter p (1380 μm) and the height h of the device, 200nm. This gives an effective growth area of only 2.76×10^{-10} m^2. Thus, total current 400nA produces average current density in the 2d regime $400 \times 10^{-9}/2.8 \times 10^{-10} = 1450$ A m^{-2}.

In the 2D regime we can now relate the total current to the expected growth front propagation rate. Faraday's law with simplifying assumptions (100% efficiency, fully dense Cu deposit) suggests that a current density of 1450 A m^{-2} drives the growth front to propagate at 53 nm s^{-1}. (Note that this result is independent of height h.)

This relationship allows us to evaluate the uniformity of the current distribution and the reproducibility of the nanoaquarium. We first note that Figure 1 shows a measured average growth front propagation rate of 55 nm s^{-1} at a current density of 400nA. This is (remarkably) close to the propagation rate expected if the current distribution were uniform. Therefore, the area imaged appears to reasonably represent the expected current density. We secondly note that scaling the current density, by carrying out a series of experiments at different total currents, even in different nanoaquarium devices, gives an average growth front propagation speed proportional to the total current (Supplementary Figure 4). This important result shows that the nanoaquarium provides reproducible current densities. Furthermore, the reciprocal $1/a = 4.4 \times 10^{-10}$ m^2 gives the effective deposition area. This is of the same order of magnitude as the 2d area (perimeter × height) calculated above.

We finally consider the local current densities that can be observed as a result of the spatial and temporal resolution afforded by liquid cell electron microscopy. We define the pointwise current density i(s, t) for a point $\boldsymbol{s}$ along the interface at time t as

$$i(\boldsymbol{s}, t) = \frac{\rho n F}{MW} \|v_N(\boldsymbol{s}, t)\|, \qquad \text{(Eq. 1)}$$

where $\|v_N(\boldsymbol{s}, t)\|$ is the magnitude of the speed of that point in the direction normal to the surface at time *t*. The pointwise normal velocity of the electrode/electrolyte interface is automatically extracted using interface tracking software as described in the text.

3. Ion transport by mechanisms other than diffusion

We have assumed that diffusion is the dominant transport mechanism, but other phenomena can also drive the motion of ions in solution. We establish below that electromigration, electroosmosis, surface conduction and advection can all be neglected in the experiment.

3.1 Fully supported electrolyte: negligible electromigration

A supporting electrolyte is used to decouple the distribution of depositing species (Cu) from the electric field. To verify that the solution is fully supported we calculate the transference number, or fraction of the migration current carried by species *j* in solution, from the equation

$$t_j = \frac{z_j^2 D_j c_j}{\sum_k z_k^2 D_k c_k}, \tag{Eq. 2}$$

where z_j is the charge, D_j is the diffusion coefficient and c_j is the concentration of ionic species j. The parameters for the species present (Supplementary Table 1) show that H^+ and SO_4^{2-} carry 97% of the current passed through the solution.

3.2 Electroosmosis

The existence of an electric double layer on the silicon nitride membrane could lead to several phenomena that result in relative motion of the fluid in the channel when an electric field is present, altering the ability of diffusion to supply reducible ions to the electrode interface. In a thin liquid layer these effects may become important: the electric double layer is on the order of 1 nm thick, while the channel is 200nm thick.

We first consider electroosmotic flow, generated when the electric field is parallel to the channel walls and ions near the electrical double layer experience electrostatic force. The resulting ion motion is transmitted to the fluid via viscous interactions and can create bulk motion within the channel. To see if this flow is important, we consider the ratio of this advective transport rate to the diffusive rate, or the Peclet number. The Peclet number takes the form of

$$Pe = \frac{V_S l_D}{D}, \tag{Eq. 3}$$

where V_S is the Smoluchowski slip velocity (or the effective velocity of ions experiencing the electroosmotic shear force), l_D is the diffusive length scale, and D is the diffusion coefficient. We can estimate the magnitude of the Smoluchowski slip velocity by

$$V_S = \frac{\epsilon \epsilon_0 E \zeta}{\mu}, \tag{Eq. 4}$$

with ϵ being the relative permeability of the solvent water, ϵ_0 the vacuum permeability, E the electric field, ζ is the zeta potential of the silicon nitride and μ the solvent viscosity. For water at 25°C, an electric field on the order of 1 V/mm, and ζ = 25 mV[2] at pH ~ 2, the Smoluchowski velocity is a few tens of μm s^{-1}. If we take the diffusive length scale to be $\sqrt{2Dt}$ with a time scale on the order of seconds, Supplementary Eq. 3 gives a Peclet number of 1.05. This means that the flow caused by electroosmosis is of the same order of magnitude as the diffusive transport rate, a fact requiring further investigation.

We use the finite element program COMSOL to evaluate electroosmotic flow effects. We consider a long, thin, rectangular channel with four impermeable walls and height 200 nm. For the ion transport we apply a flux out of the system on one of the short edges (the working electrode) and constant concentration of at the far end. The other walls are impermeable. This is implemented in COMSOL's Transport of Dilute Species Module. For fluid flow, we utilize the Laminar Flow version of the Single Phase Fluid Flow Module. The estimated Smoluchowski velocity, 30 μm s^{-1}, is implemented as a slip boundary condition on the long walls.

We run the simulation for several applied current densities, and for slip velocity zero, towards and away from the working electrode. We extract the time to reach zero concentration of the depositing ion on the working electrode as a measure of the Sand time. By comparing the results, we see very small differences in the Sand time. This implies that electroosmosis should not affect the transition to diffusion limited growth (Supplementary Table 2). However, it may still be important in other transport processes and should be considered in general liquid cell data analysis.

3.3 Surface conduction

A second transport process facilitated by the electric double layer is surface conduction, where the electric double layer acts as a "short circuit" path for the depositing ions, allowing counter ions (compared to the surface charge) to migrate along the electric field. Since silicon nitride has a positive zeta potential, SO_4^- counterions will be conducted toward the counter electrode in a process that could hinder the diffusion of Cu^{2+} to the working electrode. We estimate[3] the current I_{SC} associated with surface conduction to see how important its role is in the transport process. For applied voltage V this current is $I_{SC} = \sigma_{SC} V$, where σ_{SC} is given in the simplified Debye-Huckel limit as

$$\sigma_S = \frac{\epsilon\epsilon_0\zeta}{\lambda}, \qquad \text{(Eq. 5)}$$

with ϵ being the relative permeability of the solvent (water), ϵ_0 is the vacuum permeability, ζ is the zeta potential of the silicon nitride and λ is the Debye screening length. We estimate λ as a function of the ionic strength of the solution with

$$\lambda = \sqrt{\frac{\epsilon\epsilon_0 RT}{F^2 \sum z_i C_i}}, \qquad \text{(Eq. 6)}$$

where R is the gas constant, T is temperature, and C_i is the concentration of ionic species i with valency z_i. For the experimental parameters and voltage of 1 V, Supplementary Eq. 6 gives a current associated with surface conductance of 6.6 nA. This is at least an order of magnitude smaller than the lowest currents used during this study. This implies that transport due to surface conduction is negligible for the present study, but should be considered for low current situations where the overpotential is still order 1.

3.4 O_2 production at the counter electrode

Another possible cause of convection could be due to the production of oxygen at the Pt counter electrode. In the extreme case, suppose that O_2 is produced with 100% efficiency: the highest charge passed (400 nA, 10 s) produces 6×10^{12} O_2 molecules. If we assume this generates a gas bubble instantly and take the device pressure to be the lowest possible value (0.031 atm or the saturation pressure for water at 25°C), this O_2 would increase the total volume in the device by 8.2×10^{-12} m^3. This is less than 0.9%. For a device pressure of 1 atm, this drops to 0.05%. Thus, bulk motion in the liquid is unlikely. If we assume the gas volume is converted into flow toward the working electrode we can estimate the average fluid velocity $\bar{u}$. This is ~10^{-4} m s^{-1} for the given device dimensions. To see if the convection of ions due to this velocity is important, we calculate the Peclet number as $Pe = l\bar{u}/D$ and take the characteristic length scale of the experiment, l, to

be ~ 100 nm and the diffusion coefficient of copper to be 0.71×10^{-9} m^2 s^{-1}. This results in Pe ~ 10^{-2}, implying diffusion is still the dominant form of mass transfer.

4. *Ex situ* Hull Cell experiments with Pb additive

To evaluate the effect of Pb on growth we examine *ex situ* the morphology as a function of current density. We employ a simple flat plate Hull Cell[3], Supplementary Figure 5, to examine the onset of ramified growth in the absence and presence of the additive. 200 mA total current was applied for 300 s. This results in a current density that depends on position[7] according to

$$\frac{i(x)}{i_{ave}} = \frac{(x/L_H)^{1.2733}}{(1-x/L_H)^{0.359}}(1.733 - 0.763\,x/L_H) \qquad \text{(Eq. 7)}$$

Here the average current density $|i_{ave}|$ is 20 A m^{-2}; L_H and x are defined in Supplementary Figure 5 and $i(x)$ is the current density at position x. Post growth, we estimate roughness by light microscopy, by determining the location at which the asperities are greater than the depth of field (~1.5µm). For the plain solution, this occurred at position 2.2±0.4mm, at which the current density was 44 A m^{-2}. For the solution with Pb this occurred at 0.6±0.4mm, with current density 74 A m^{-2}, or 1.7 times greater. This supports the *in situ* finding that Pb suppresses asperities up to a higher current density than the plain solution.

Supplementary References

1. L. Bousse and S. Mostarshed, The zeta potential of silicon nitride thin films, *Journal of Electroanalytical Chemistry and Interfacial Electrochemistry* **302**, 269–274, 1991.
2. J.-H. Han, E. Khoo, P. Bai and M. Z. Bazant, Over-limiting current and control of dendritic growth by surface conduction in nanopores, *Scientific Reports* **4**, 7056, 2014.
3. A. C. West, Electrochemistry and Electrochemical Engineering: An Introduction. CreateSpace Independent Publishing Platform, 2012.
