# Supplementary material for: Nanoscale evolution of interface morphology during electrodeposition
Source: Nat Commun. 2017 Dec 19;8:2174. doi: 10.1038/s41467-017-02364-9 (PMC5736733; doi:10.1038/s41467-017-02364-9)
Supplement: Supplementary file 2 — Description of Additional Supplementary Files [file 41467_2017_2364_MOESM2_ESM.pdf]

Description of Additional Supplementary Files

File Name: Supplementary Movie 1
Description: **M1 (images in Figure 1a)**. Deposition at total current 400nA showing a slow increase followed by rapid increase in roughness. The region is shown over a longer time than the deposition analyzed to show electron beam effects after 15s. Speeded up by 2×, total duration 18 s.

File Name: Supplementary Movie 2
Description: **M2 (images in Figure 2a)**. Pulsed deposition showing a slow increase then saturation in roughness. The first cycle contains an electrode stripping / cleaning cycle of 15s after which 75nA was cycled on and off for 10s periods. Short pauses occur after every 5 cycles to restart the potentiostat due to limitation in our programming. This results in the jumps in the video after every 5th cycle. Video speeded up by 20×, total duration 515s. Note that the time labels on this data differ from those in Figure 64 of Ref. [40], due to a software error in the frame rate used and also a change from counting only the on-time to including on and off time in Figure 2 to be consistent with the diffusion calculations. Furthermore, Ref. [40] included some profiles obtained at longer times for which the model is not expected to apply and that are truncated here.

File Name: Supplementary Movie 3
Description: **M3 and M4 (calculations in Figures 1c and 2c respectively and in Supplementary Figure 1).** The Cu^{2+} concentration vs. time and distance from the growth front, calculated for the experimental parameters in Videos M1 and M2 respectively. In M3 this was 1550 A m^{-2} for 20s and in M4 this was 35 A m^{-2} for 10s then 0nA for 10s, first 5 cycles (100s total time). M3 is slowed by 10× to show details of the first 1s. M4 is speeded up by 10×. Plots obtained from Videos M3 and M4 and from a calculation using the current density for experimental Video M6 are shown in Supplementary Figure 1.

File Name: Supplementary Movie 4
Description: **M5 (images in Figure 4a)**. Pulsed deposition showing slow increase in roughness. Each cycle consists of 400nA for 1s then 0nA for 5s. This experiment used a two-electrode configuration giving large measured potential. A bubble became visible in the field of view after six cycles and changed the liquid geometry (electrolyte thickness). Speeded up by 4×, total duration 24 s.

File Name: Supplementary Movie 5
Description: **M6 (images in Figure 5a)**. Deposition from an electrolyte containing saturated $PbSO_4$. 300nA was applied for 10s, 0nA for 10s, repeated, showing no increase in roughness. Speeded up by 2×, total duration 18 s.
